# Lineage Replacement Associated with Fitness Gain in Mammalian Cells and *Aedes aegypti*: A Catalyst for Dengue Virus Type 2 Transmission

**DOI:** 10.3390/microorganisms10061100

**Published:** 2022-05-26

**Authors:** Cheong Huat Tan, Hapuarachchige Chanditha Hapuarachchi, Li Kiang Tan, Pei Sze Jeslyn Wong, Mei Zhi Irene Li, Wing Yan Wong, Lee Ching Ng

**Affiliations:** 1 Environmental Health Institute, National Environment Agency, 11 Biopolis Way, #06-05-08, Singapore 138667, Singapore; tan_cheong_huat@nea.gov.sg (C.H.T.); tan_li_kiang@sfa.gov.sg (L.K.T.); jeslyn_wong@nea.gov.sg (P.S.J.W.); irene_li@nea.gov.sg (M.Z.I.L.); wong_wing_yan@nea.gov.sg (W.Y.W.); ng_lee_ching@nea.gov.sg (L.C.N.); 2 School of Biological Sciences, Nanyang Technological University, 60 Nanyang Drive, Singapore 637551, Singapore

**Keywords:** Dengue virus 2, *Aedes aegypti*, clade replacement, fitness, transmission, epidemic potential

## Abstract

Shifting of virus serotypes and clade replacement events are known to drive dengue epidemics. However, only a few studies have attempted to elucidate the virus attributes that contribute to such epidemics. In 2007, Singapore experienced a dengue outbreak affecting more than 8000 individuals. The outbreak ensued with the shuffling of dominant clades (from clade I to clade II) of Dengue virus 2 (DENV-2) cosmopolitan genotype, at a time when the *Aedes* premise index was significantly low. Therefore, we hypothesized that clade II had higher epidemic potential and fitness than clade I. To test this hypothesis, we tested the replication and apoptotic qualities of clade I and II isolates in mammalian cells and their ability to infect and disseminate in a field strain of *Ae. Aegypti.* Our findings indicated that clade II replicated more efficiently in mammalian cells than clade I and possessed higher transmission potential in local vectors. This could collectively improve the epidemic potential of clade II, which dominated during the outbreak in 2007. The findings exemplify complex interactions between the emergence, adaptation and transmission potential of DENV, and testify the epidemiological importance of a deeper understanding of virus and vector dynamics in endemic regions.

## 1. Introduction

Dengue is the most widespread arboviral disease, with an estimated annual burden of approximately 390 million infections [1]. The annual global cost resulting from an estimated 58.4 million symptomatic cases is US$ 8.9 billion [2]. Dengue virus (DENV), the causative agent of dengue, is a positive-sense, single-stranded RNA virus consisting of four immunologically related but distinct serotypes (DENV-1–4). DENV is primarily spread by *Aedes aegypti* and less efficiently by *Aedes albopictus* [3].

Singapore is hyperendemic to dengue, where all serotypes cocirculate in different proportions [4,5]. Epidemics have occurred in a cyclical pattern over the last two decades, driven by DENV-1 and -2 [6]. In 2005, Singapore experienced an unprecedented outbreak, causing 14,000 reported cases and 14 deaths [7]. This was followed by another outbreak three years later with more than 8000 reported cases and 24 deaths [8]. The subsequent epidemic from 2013–2014 caused more than 40,000 cases [5]. The latest outbreak, which was the worst recorded so far, inflicted ~51,000 cases during the 2019–2020 period. All these epidemics/outbreaks witnessed dominant serotype shifts; from DENV-2 to DENV-1 in 2005 and 2013 and vice versa in 2007 and 2019. The DENV-2 switch leading to the outbreak in 2007 coincided with a clade replacement event in the cosmopolitan genotype that resulted in the emergence of a new clade (clade II) at the expense of a previously circulating clade (Clade I) of DENV-2 [4]. Clades I and II phylogenetically branched into two distinct groups that can be distinguished by nine fixed amino acid substitutions across the virus genome.

Lineage turnover or clade replacement events within serotypes is a common phenomenon in dengue epidemiology [9,10,11,12,13,14]. Although lineage turnover has been shown to play an important role in shaping the disease outcome and epidemic risk [15,16], underlying evolutionary forces effecting viruses to correlate with disease transmission remain unclear [17]. Even though the clade replacement and lineage dominance within DENV serotypes can be due to nonviral factors [18], such events are also attributable to increased viral fitness in both humans and vectors, implying that a host-driven selection plays an important role in shaping virus evolution [11,19]. Vector-driven selection is a natural force that drives the evolution of arboviruses by selecting lineages or genotypes that are better suited for transmission by vectors [11,12]. Previous evidence testifies that the selection process may be enhanced by the emergence of DENV strains with high host and vector adaptability, imposing a tremendous impact on their epidemic behavior [16].

Clade II dominated during the outbreak in 2007, which unfolded at a time when the *Aedes* premise index (0.68%) was significantly lower (1.7–2.2% from 2002 to 2004) than that recorded during the preceding, non-outbreak years during which clade I circulated. Therefore, we hypothesized that clade II possesses superior epidemic potential to clade I. To test this hypothesis, we investigated the replication kinetics of clade I and II isolates in myelogenous leukemia cell line K562 as a surrogate for virus fitness in mammalian cells. We also compared the ability of the two clades to infect and disseminate in local *Ae. aegypti* mosquitoes. We discuss the differences in fitness of clades I and II in mammalian cells (in vitro) and vector (in vivo) that could have contributed to the outbreak in 2007–2008.

## 2. Materials and Methods

### 2.1. Cell Lines

Chronic myelogenous leukemia cell line K562 (courtesy of Asst. Prof. Justin Jang Hann Chu, National University of Singapore) and baby hamster kidney cell line (BHK-21, courtesy of Novartis Institute of Tropical Diseases, Singapore) were scaled up in RMPI growth medium (Sigma-Aldrich Corp., St. Louis, MO, USA) supplemented with 10% heat-inactivated Fetal Bovine Serum (FBS) (Thermo Scientific, Oxford, UK), 2 mM L-glutamine, 100 mM penicillin/streptomycin, 10 mM HEPES and 1 mM sodium pyruvate (Invitrogen, Carlsbad, CA, USA). Both cell lines were cultured at 37 °C with 5% CO_2_.

*Aedes albopictus* cell line (C6/36, ATCC CRL 1660) was maintained at 28 °C in Leibovitz’s L-15 growth medium (Invitrogen, USA), supplemented with 10% heat-inactivated FBS, 2 mM L-glutamine, 100 mM penicillin/streptomycin (Invitrogen, USA). Maintenance media used for the virus-related work was prepared as described above but was supplemented with 3% FBS.

### 2.2. Virus Isolates

DENV2 viruses used in the present study (Table 1) were isolated from patient sera obtained from residual diagnostic samples received by the Environmental Health Institute (EHI) Diagnostics. Virus isolates were selected from a pool of clades I and II viruses, classified based on the nine amino acid substitutions distinguishable between the two lineages [4,15]. 

Each isolate was obtained by inoculating 25 µL of respective sera into C6/36 cell line that was maintained in Leibovitz’s L-15 medium (Invitrogen, USA), supplemented with 3% heat-inactivated FBS, 100 U/mL penicillin, 100 µg/mL streptomycin and 2 mM L-glutamine at 33 °C for 4–5 days. Each isolate was subjected to three passages. Virus supernatants were stored at −80 °C and titrated by plaque assay [20]. Genotype of each virus was characterized based on the envelope gene phylogeny as described previously [4].

### 2.3. Ethics Statement

Virus strains used in the present study were originally isolated from patient sera obtained after written informed consent. The sera were collected as part of the dengue surveillance program in Singapore. The ethics approval was granted by the National Environment Agency Institutional Review Board (IRB 0.0003.1). All experiments were performed in accordance with IRB guidelines and regulations.

### 2.4. Complete Genome Sequencing of Virus Isolates

The complete genome sequences of all isolates were generated as described elsewhere [21]. Nucleotide sequences were assembled using the Lasergene package version 8.0 (DNASTAR Inc., Madison, WI, USA). Contiguous sequences were aligned against DENV-2 complete polyprotein sequences (n = 1.616) available in the GenBank database at the time of writing and the unique amino acid differences were deduced by using the BioEdit v7.0.9.0 software [22]. The analysis also included clade I (n = 13) and clade II (n = 15) polyprotein sequences, reported earlier [4], in order to confirm the “fixed” mutation patterns in each group of viruses.

### 2.5. Phylogenetic Analysis

The whole polyprotein-based maximum likelihood tree was constructed in MEGA7 program [23], using the general time reversible model with gamma distribution (Γ5) and invariant sites (GTR+G+I). The robustness of the original tree was tested with 1000 bootstrap replications. The dataset included 28 whole polyprotein sequences of clade I and clade II strains reported in Singapore from 2004 to 2009. An additional 63 sequences retrieved from the GenBank database were included to represent different genotypes of DENV-2.

### 2.6. Secondary Structure Analysis of 3′ UTR

The secondary structures of the 3′ untranslated region (UTR) of different DENV-2 genotypes were predicted using the MFOLD web server at http://mfold.rna.albany.edu; (accessed on 15 March 2022) under standard conditions (37 °C) [24].

### 2.7. Infection of K562 Cells

To determine the in vitro infection rates and growth kinetics of clade I and II viruses, a time course experiment was conducted. K562 cells were infected with each virus strain at a multiplicity of infection (M.O.I) of 5 in suspension and were incubated at 37 °C for 90 min. Cell suspensions were rocked gently at every 20 min interval to achieve even virus-cell contact. A cell-only suspension was used as a negative control. Unbound virus particles were removed by washing the cells with phosphate-buffered saline (PBS) twice and centrifuged at 3000 rpm for 10 min. Cell pellets were resuspended with fresh medium and seeded in 24-well plates at a density of 1 × 10^5^ cells/well. Supernatants were collected daily for six days and were stored in aliquots at −80 °C. Culture supernatants collected from 0 hr post-infection (hpi) till 6th day post-infection were used for the viral quantitation by plaque assay and quantitative real-time RT-PCR. Cells harvested at 24 and 48 hpi were immuno-stained and were subjected to flow cytometry analysis to determine the percentage of cells that expressed viral antigens and the percentage of apoptotic cells. All in vitro experiments were carried out in triplicates.

### 2.8. Quantitation of Viral RNA by Real-Time RT-qPCR

Viral RNA was extracted by using the QIAGEN QIAamp viral RNA minikit (QIAGEN, Germany) according to the manufacturer’s recommendations. RNA copy numbers in supernatants were determined by using a probe-based RT-qPCR assay. Each reaction was performed in a total volume of 20 μL containing 1× Quantitect^®^ probe RT-PCR kit master mix (QIAGEN, Hilden, Germany), 0.4 μM of forward and reverse primers, 0.15 μM probe, 5 μL of template and nuclease-free water. Primers and probe sequences used were Den F2 (5′-AAA CAG CAT ATT GAC GCT GGG A-3′), Den R3 (5′-GGC GYT CTG TGC CTG GAW TGA TG-3′) and probe (5′FAM-AGA CCA GAG ATC CTG CTG TCT C-3′BHQ1). PCR conditions were as follows: holding temperature at 50 °C for 30 min and 95 °C for 15 min, followed by 45 cycles of 94 °C for 15 s, 57 °C for 60 s and 57 °C of 60 s. RNA copy numbers were quantified based on a standard curve created by using the in vitro transcribed RNA of a known concentration serially diluted from 2 × 10^8^ to 2 × 10^10^ copies.

### 2.9. Detection of Intracellularly Expressed Virus Antigens by Flow Cytometry

Cells harvested at 24 and 48 hpi were stained intracellularly by using a BD Cytofix/CytopermTM Fixation/Permeabilization kit (BD Bioscience, Franklin Lakes, NJ, USA) as per the manufacturer’s protocol. Briefly, harvested cells were washed with filtered 1× PBS by centrifugation at 1500 rpm for 5 min. Cell pellets were then fixed and permeabilized in 250 μL of Fixation/Permeabilization solution for 15 min at 4 °C. After washing with 1× Permeabilization/Wash buffer, fixed cells were stained first with intracellular monoclonal antibody (HB112 [D1 4G2, ATCC]), followed by 2 μL of secondary antibody (goat anti-mouse IgG (H+L)-FITC conjugated, Millipore, CA, USA) at 1:30 dilution for 30 min at 4 °C in the dark. Unbound antibodies were washed with 1× Permeabilization/Wash buffer. One microliter of IgG2 (mouse Ig2a-FITC, Miltenyi Biotec, Gaithersburg, MD, USA) at 1:10 dilution was used as the isotype control. Stained cells were fixed with 500 μL of 1% formaldehyde/PBS by incubating at 4 °C in the dark for at least 10 min. After removing formaldehyde, cells were suspended with staining media (DPBS with 1% heat-inactivated FBS) before proceeding to flow cytometry acquisition. The percentage of DENV-infected cells was determined using FACS Canto II (Becton Dickinson, Franklin Lakes, NJ, USA).

### 2.10. Analysis of Apoptotic Cells by Flow Cytometry

Virus-infected and control cells harvested at 24 and 48 hpi were washed twice with 1× cold PBS, stained with APC Annexin V (BD PharmingenTM, BD Bioscience, USA) in 1× binding buffer (1.4 M NaCl, 25 mM CaCl2 and 0.1 M Hepes) in dark at room temperature for 15 min according to manufacturer’s instructions. All cells were acquired within an hour using MACSQuant Analyzer (Miltenyi Biotec, Bergisch Gladbach, Germany), with 5000 gated events for each analysis.

### 2.11. Mosquito Colonies

The local strain of *Ae. aegypti* used in the study was derived from eggs collected as part of ovitrap surveillance activities as previously described [25]. Mosquitoes were colonized under the standard insectary conditions as described elsewhere [25,26]. The colony was maintained for up to three generations (F3) to obtain an adequate number of mosquitoes for the experiments.

### 2.12. Oral Infection of Mosquitoes

Five to seven-day old female *Ae. aegypti* mosquitoes were starved for 24 h prior to the infectious blood meal. Each blood meal consisted of 1:1 washed specific-pathogen free swine blood (A*star Biomedical Resource Centre, Singapore) and fresh virus suspension. Adenosine 5′-Triphosphate (Fermentas, Waltham, MA, USA), at a final concentration of 3 mM, was used as a phagostimulant. The virus titer in each blood meal was 5.95 Log_10_TCID_5__0_/mL. The feeding was carried out for 30 min using a Hemotek membrane feeding system (Discovery Workshops, Trowbridge, UK) set at 37 °C. All mosquitoes were cold anesthetized, and fully engorged females were transferred to new paper cups covered with net and maintained in an environmental chamber (Sanyo, Osaka, Japan) at 29 °C and 70–80% RH with a 12 h/12 h light:dark cycle. These mosquitoes were fed with 10% sugar/vitamin B complex ad libitum.

Oral infection and processing of mosquitoes were carried out in the arthropod containment level 2 facility at the Environmental Health Institute, Singapore.

### 2.13. Processing of Mosquitoes

Ten blood-fed mosquitoes were sampled at seven timepoints post-infection for each virus isolate: daily from day 3 to 7, on day 10 and day 14. The total number of mosquitoes processed was 140. The midgut and salivary glands of each mosquito were dissected and individually homogenized in Medium 199 (Thermo Fisher Scientific, Waltham, MA, USA) supplemented with amphotericin B (Sigma Aldrich, USA), using a MM 301 mixer mill (Retsch, Haan, Germany).

### 2.14. Virus Titration by Using 50% Tissue Culture Infectious Dose (TCID50) Assay

Viral titers of midguts and salivary glands obtained from blood-fed mosquitoes were determined by 50% tissue culture infectious dose assay in Vero cells as described previously [27]. Briefly, samples were titrated in a 10-fold serial dilution in 96-well microtiter plates and incubated with 5% CO_2_ at 37 °C for seven days. The cells were then fixed with ice-cold acetone: methanol (1:1) mixture for at least 20 min at −20 °C. After removing the fixative, cells were dried prior to immunostaining. To prevent nonspecific binding of the primary antibody, cells were rehydrated with 1% normal goat serum (Merck, Rahway, NJ, USA) in 1× phosphate-buffered saline (PBS) for 10 min. The solution was removed and 1:400 mouse IgG2a anti-dengue Ab (ICL, St. Louis, MO, USA) diluted in 1% normal goat serum (Merck, USA) in 1× PBS was added to each well and incubated for 30 min. After two washes in 1× PBS, wells were incubated for another 30 min with 1:500 goat anti-mouse horseradish peroxidase-conjugated secondary Ab (Dako, Santa Clara, CA, USA) diluted in 1% normal goat serum (Merck, USA) in 1× PBS, followed by two washes with 1× PBS. VIP substrate (Vector Laboratories, Burlingame, CA, USA) was then added to each well and incubated for 5 min before washing with water once. After drying, each well of the microtiter plates was examined using an inverted microscope (40× objective; Olympus Corp., Tokyo, Japan). A well was scored positive if a purple product was observed compared to the uninfected control cells. All virus concentrations were calculated using the Spearman–Kärber method and expressed in Log_10_ TCID_50_/mL [28].

### 2.15. Data Analysis

All mosquitoes with midgut infections were considered to be infected, whereas mosquitoes with salivary gland infection were considered to have disseminated infection. Infection rate was defined as the proportion of DENV2-positive midgut, while dissemination rate was defined as the proportion of DENV2-positive salivary glands out of total number of mosquitoes processed. Mann–Whitney U test was used to determine the difference in viral titers between clade I and clade II in mosquito midguts and salivary glands. *p*-values of <0.05 were considered statistically significant. Statistical analyses were conducted using the MedCalc for Windows (MedCalc software, Oostende, Belgium).

Student’s *t*-test was used to compare virus titers, RNA copy numbers, infection rates and apoptosis rates between the two clades. Data was expressed as means + standard errors of the means (S.E.M). Correlation analysis was performed using R version 2.15.1 software package [29]. Pearson Correlation coefficients ^®^ were calculated through function ccf, and linear regressions were conducted with function lm. *p*-values of <0.05 were considered statistically significant. Statistical analyses were conducted using the Minitab16 software (Minitab LLC, State College, PA, USA) and Microsoft Excel (Microsoft, Redmond, WA, USA).

## 3. Results

### 3.1. Two Clades Were Genetically Distinguishable by a Unique Substitution Pattern

**Figure 1:**
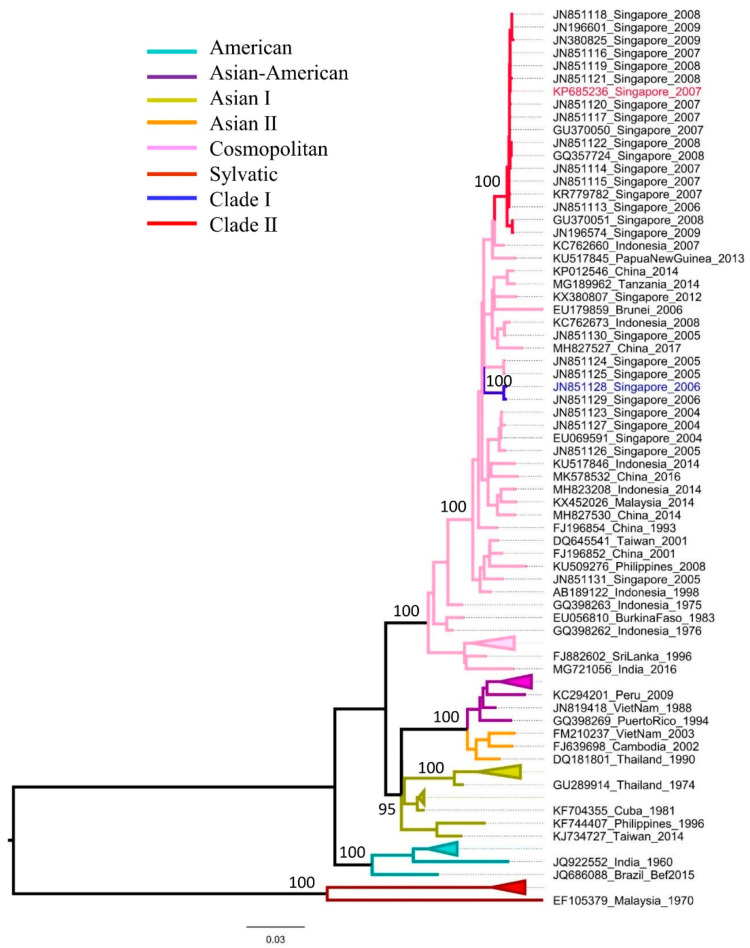
Phylogenetic analysis to illustrate the genetic distinction between clades I and II. The whole polyprotein-based maximum likelihood tree was constructed in the MEGA7 program [23]. The dataset included 28 whole polyprotein sequences of clade I and clade II reported in Singapore from 2004 to 2009 and 63 sequences retrieved from the GenBank database to represent different genotypes of DENV-2. Genotypes and clades are shown in different colors as noted in the legend. The taxa names of isolates used in the present study are colored in each clade. The numbers on branches show bootstrap support values.

Complete polyprotein-based phylogenetic analysis demonstrated that clades I and II were distinct with strong bootstrap support (Figure 1). This is in agreement with a previous study that showed the genetic distinction between these two clades based on envelope gene sequences [15]. The mean nucleotide divergence between clades I and II was 0.021 (range: 0.018–0.022). This corresponded to a difference of 213 nucleotide substitutions between the two clades in their complete polyprotein sequences (10,176 nucleotides). The nucleotide divergence among clade II sequences (n = 17) was 0.004, whereas that of clade I sequences (n = 2) was 0.002. Comparative sequence analyses confirmed that clade II isolates were distinguishable from clade I by a unique combination of nine amino acid substitutions. Of these, four substitutions were in the structural polyprotein (E-I61V, M-V36I, M-A71T, M-T75A) and the remaining were in the nonstructural polyprotein (NS1-S80T, NS2A-T119S, NS4A-R22K, NS5-T638K and NS5-A648I/M). Because of the involvement of untranslated regions (UTR) in flavivirus replication and vector adaptation shown in other studies [30], we also compared the nucleotide differences of UTRs between clade I and II isolates. Two clades were indistinguishable in the 5′ UTR, but were different by three nucleotide substitutions (A10274G, C10387T and T10389C) in the variable region of 3′ UTR. Of these, A10274G was “fixed” in clade II isolates, whereas the other two substitutions were found in clade I viruses. The predicted secondary structure demonstrated different stem loop IV (SL-IV) [31,32] arrangements between clade I and II isolates (Additional Figure 1), but the remaining regions of 3′ UTR were identical.

### 3.2. Clade II Demonstrated Higher Replication Efficiency than Clade I in Mammalian Cells

**Figure 2:**
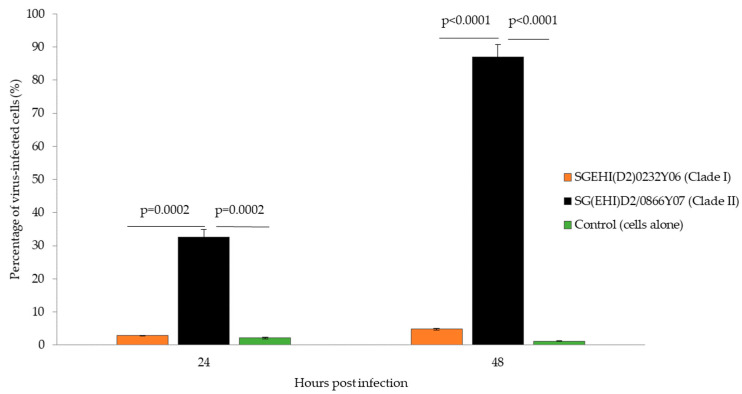
Percentage of K562 cells infected by DENV-2 strains. Cells infected with clade I and II isolates (M.O.I = 5) were collected between 24 and 48 hpi and subjected to flow cytometry with MAbs against the envelope protein. The data show means of triplicates and are representative of two independent experiments. Statistically significant observations are shown with *p*-values. Error bars indicate ± S.E.M.

We investigated the replication efficiency of clade I and II viruses in mammalian cells by establishing their growth kinetics in K562 cells. K562 is a chronic myelogenous leukemia human cell line [33] and exhibits characteristics of monocytes [34]. The infected K562 cells and control cells harvested at 24 and 48 hpi were subjected to flow cytometry analysis to determine the percentage of infected cells that expressed viral antigens. Overall results showed that clade II achieved higher infection rates than clade I (Figure 2). At 24 hpi, SG(EHI)D2/0866Y07 (clade II) produced an infection rate of 32.6% as compared to the clade I isolate (SGEHI(D2)0232Y06; 2.86%; *p* = 0.0002). At 48 hpi, infection rates of the clade II isolate [SG(EHI)D2/0866Y07] achieved 87.06%, which was approximately 18 times faster than the clade I isolate SGEHI(D2)0232Y06 (4.8%; *p* = 0.0001).

**Figure 3:**
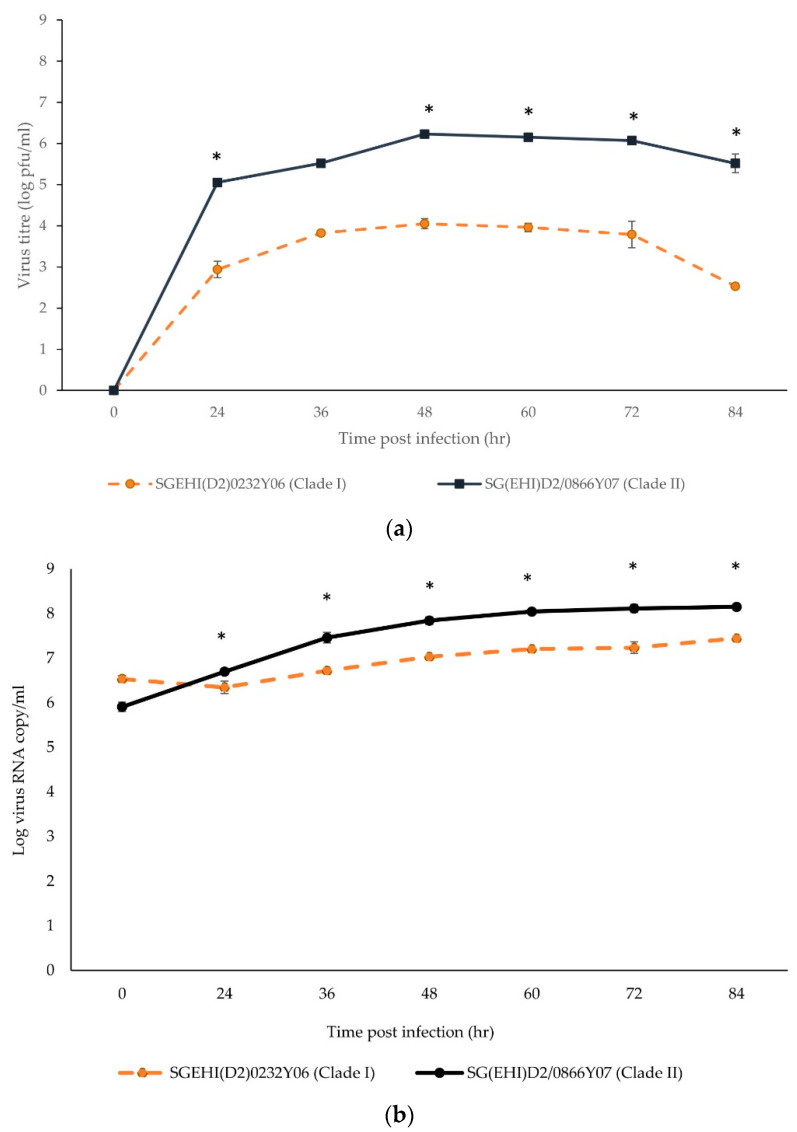
Growth kinetics of DENV-2 strains in K562 cells. Cells infected with clade I and II isolates (M.O.I = 5) were incubated for six days. The data show means of triplicates and are representative of two independent experiments. (**a**) Virus titers in supernatants were quantitated by plaque assay and expressed as logPFU/mL. Asterisk represents *p* < 0.002. (**b**) Viral copy numbers in supernatants were determined by RT-PCR. Asterisk represents *p* < 0.0.05. Error bars indicate ± S.E.M.

The clade II isolate achieved the peak virus titers between 48 hpi and 72 hpi (Figure 3a) before reaching a plateau. The clade II isolate was more competent (~1–2 log increase) than the clade I isolate at 24 hpi and produced significantly higher infective viral progenies from 24 hpi to 72 hpi (*p* < 0.05). At 36 hpi, the virus titer of SG(EHI)D2/0866Y07 (clade II) was greater than the clade I isolate by almost 2 logs (*p* ≤ 0.002). Even though a high amount of viral RNA was detected at 0 hrs due to incomplete washing of the unbound virus particles, SG(EHI)D2/0866Y07 (clade II) showed significantly higher viral RNA copy numbers as compared to the clade I isolate from 24 hpi to 72 hpi (*p* ≤ 0.046) (Figure 3b). This demonstrated that the clade II isolate generates consistently higher virus progeny than the clade I isolate when actively replicating RNA is likely to be measured. Overall, the clade II isolate demonstrated better replication efficiency than the clade I isolate in mammalian cells.

### 3.3. Clade II Produced Lower Percentage of Apoptotic Cells

**Figure 4:**
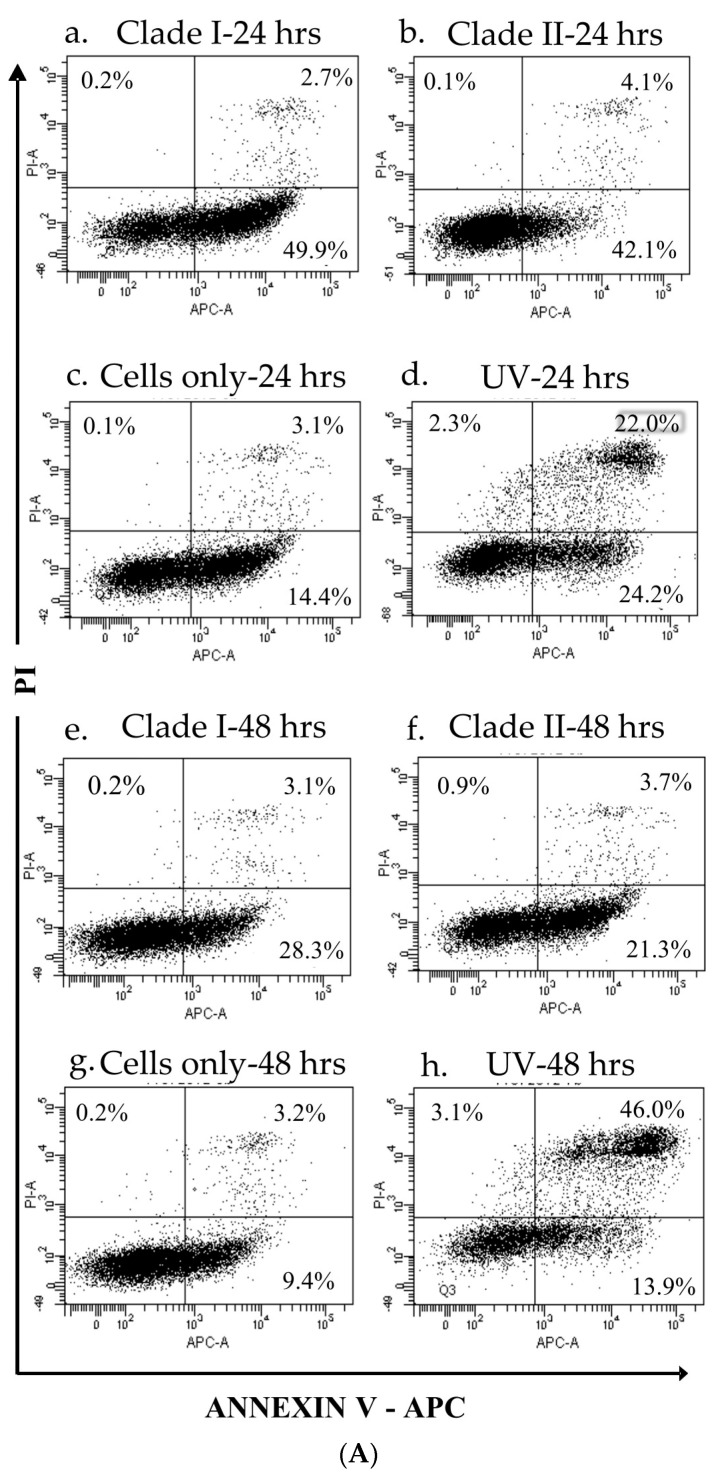

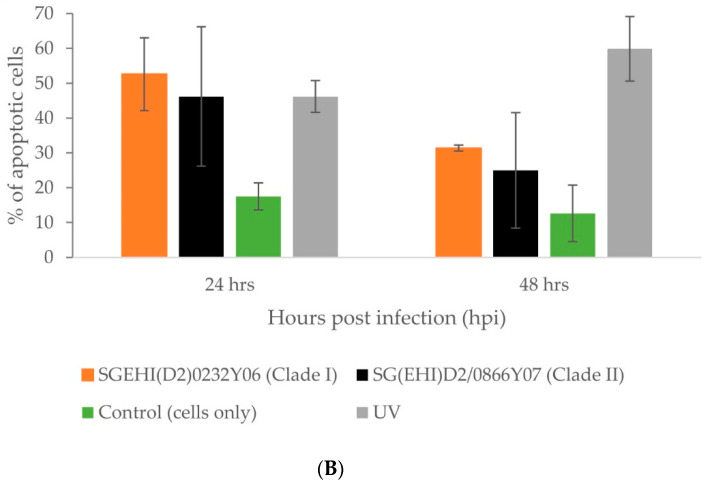
Apoptotic profiles of K562 cells infected by DENV−2 isolates. Cells infected with clade I and II isolates (M.O.I. = 5) were collected at 24 and 48 hpi, stained with Annexin-V/PI and analyzed using flow cytometer (**A**). Dot plots show the apoptotic profiles of a representative set of K562 cells at 24 hpi (**a**–**d**) and 48 hpi (**e**–**h**). Lower left quadrant represents cells negative for both Annexin-V and PI. Lower right quadrant represents Annexin-V-positive cells (early apoptotic cells). Upper left quadrant represents PI-positive, but Annexin-V-negative cells. Upper right quadrant represents cells positive for Annexin-V and PI (necrotic cells). Clade I (SGEHI(D2)0232Y06) is shown in panels (**a**,**e**). Clade II (SG(EHI)D2/0866Y07) is shown in panels (**b**,**f**). Cells alone (**c**,**g**) and cells subjected to UV treatment (**d**,**h**) were included as negative and positive controls, respectively. The data are from a single experiment performed using technical triplicates. The mean percentage of cells stained/non-stained with Annexin-V and/or PI among the total number of cells used in triplicates are shown in the quadrants of each panel. (**B**) Percentage of apoptotic cells. The data show the cumulative values of upper (Annexin-V-positive necrotic cells) and lower (early apoptotic cells) right quadrants of each panel in (**A**). The differences are not significant between clade I (SGEHI(D2)0232Y06) and clade II (SG(EHI)D2/0866Y07) isolates at 24 and 48 hpi. Error bars indicate ± S.D.

Flow cytometry analysis showed that clade II induced a lower percentage of apoptotic cells than the clade I isolates at 24 hpi and 48 hpi (Figure 4A,B), but the differences were not statistically significant. The apoptotic rate of the clade I isolate showed a strong positive correlation with the infection rate (Pearson correlation coefficient = 0.75, *p* < 0.001) during 48 hpi, whereas the clade II isolate demonstrated a weak correlation between the two parameters (Pearson correlation coefficient = 0.45, *p* = 0.01).

### 3.4. Clade II Demonstrated Earlier Dissemination than Clade I in Ae. aegypti

**Figure 5:**
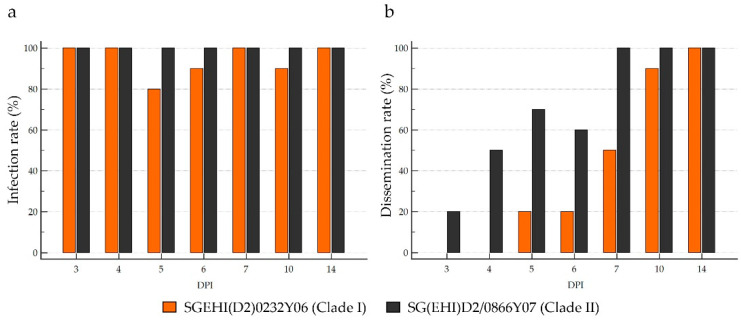
Infection and dissemination rates of clade I and II isolates in *Ae. aegypti*. Ten mosquitoes were sampled for each virus isolate per sampling day. Infection rate (**a**) is the proportion of DENV2-positive midgut, while dissemination rate (**b**) is the proportion of DENV2-positive salivary glands out of total number of mosquitoes tested.

A total of 140 *Ae. aegypti* mosquitoes orally infected with clade I (n = 70) and clade II (n = 70) isolates were processed. All mosquitoes developed midgut infection at 3-day post-infection (dpi), regardless of the infecting virus strain, indicating high susceptibility of local *Ae. aegypti* mosquitoes to DENV-2 cosmopolitan genotype (Figure 5a). The midgut infection rate of the clade II isolate was 100% for all sampling days. Clade I isolate also achieved a 100% midgut infection rate on all sampling days except on 5-, 6- and 10-dpi, but the differences on those days were not statistically significant.

The clade II isolate was evident in the salivary glands of the infected mosquitoes (3-dpi) earlier than in the clade I isolate (5-dpi) (Figure 5b). By 7-dpi, the salivary glands of all mosquitoes infected with the clade II isolate were positive. In contrast, the clade I isolate achieved a 100% salivary gland dissemination rate on 14-dpi. This was despite the clade I isolate achieving a 100% midgut infection rate as early as 3-dpi, suggesting a faster virus dissemination of the clade II isolate than the clade I isolate.

### 3.5. Clade II Achieved Higher Virus Titers than Clade I in Ae. aegypti Salivary Glands

**Figure 6:**
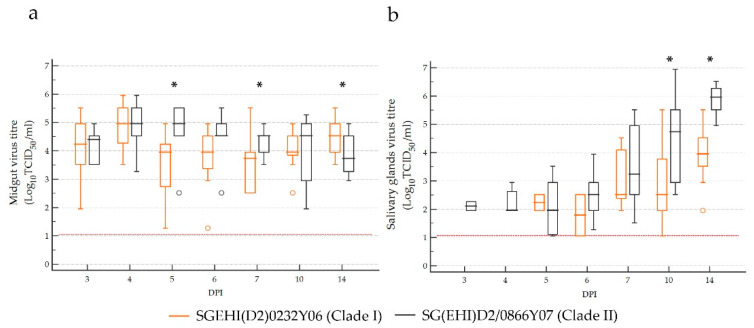
*Ae. aegypti* midgut (**a**) and salivary gland (**b**) virus titers at different timepoints following oral infection with clade I and II isolates. The central box represents the values from the lower to upper quartile (25 to 75 percentile). The middle line represents the median. The horizontal line extends from the minimum to the maximum value, excluding far-out values, which are displayed as separate points. Asterisks indicates a *p*-value of <0.05 by the Mann–Whitney U test between the two clades. The limit of detection is represented by the red dotted line.

The midgut viral titers of the clade I isolate ranged from 1.27 to 5.95 Log_10_TCID_50_/_mL_, while those of clade II ranged from 1.95 to 5.95 Log_10_TCID_50_/mL, without an obvious trend over the sampling period (Figure 6a). Despite being fed with similar titers (5.95 Log_10_TCID_50_/mL), the clade II isolate achieved significantly higher midgut viral titers than the clade I isolate on 5- and 7-dpi. However, the clade I isolate reached significantly higher midgut titers than the clade II isolate on 14-dpi, suggesting that clade I is as competent as clade II in regard to midgut infection even though its replication is delayed.

The salivary gland viral titers of the clade I isolate ranged between 1.06 to 5.52 Log_10_TCID_50_/mL, while those of the clade II isolate were between 1.06 to 6.95 Log_10_TCID_50_/mL (Figure 6b). Virus titers of both clades demonstrated an upward trend from 5-dpi. Although the median midgut infection titers were not significantly different on 3-dpi (clade I–4.24 Log_10_TCID_50_/mL, clade II–4.40 Log_10_TCID_50_/mL) and 4-dpi (clade I and II–4.95 Log_10_TCID_50_/mL), only the clade II isolate reached detectable salivary gland titers as early as 3-dpi (1.95 to 2.27 Log_10_TCID_50_/mL). The median salivary gland titer of the clade II isolate was consistently higher than the clade I isolate from 6-dpi, and the differences were statistically significant on 10- and 14-dpi.

## 4. Discussion

The transmission cycle of DENV consists of a vertebrate (human) host and an invertebrate (primarily the *Ae. aegypti* mosquito) host. Consequently, the transmission success of DENV at a particular location primarily depends on the virus’s ability to evade population immunity in humans as well as to gain fitness in local vectors [35,36,37]. This is achieved through evolutionary dynamics and adaptations to host genotypes [38,39]. Even though DENV populations are highly diverse and dynamic [40], only a few virus lineages that are able to overcome these challenges generally establish sustained transmission and cause outbreaks [18]. These lineages become dominant during the outbreak transmission, demonstrating serotype/genotype/clade replacements [10,11,41]. This was also evident during the 2007 outbreak in Singapore, which was associated with a switch in the predominant serotype from DENV1 to DENV2, along with a clade replacement (from clade I to II) within the DENV2 cosmopolitan genotype [15]. In the present study, we investigated whether the superior epidemic potential of clade II was due to its better fitness in mammalian cells and *Ae. aegypti* mosquitoes than clade I.

DENV fitness is generally reflected by its ability to replicate and disseminate in hosts. The findings showed marked differences in the efficiency of replication and dissemination between clade I and II isolates. Notably, the clade II isolate achieved higher salivary gland titers than the clade I isolate in *Ae. aegypti*. Clade II demonstrated a shorter extrinsic incubation period (EIP), as the virus disseminated more efficiently to reach the salivary glands as early as 3-dpi compared to clade I, which spent 5-dpi. Moreover, the clade II isolate achieved a 100% salivary gland infection rate in one week after oral infection compared to two weeks for clade I isolate. Consequently, *Ae. aegypti* infected with clade II had a higher probability of being infectious within a shorter period than those infected with clade I. Furthermore, clade II isolate demonstrated higher virus titers than clade I in mammalian cells within 72 hpi. The clade II isolate induced less apoptosis than clade I in the mammalian cells. Even though the differences were not statistically significant, this trend was consistent at 24 hpi and 48 hpi, suggesting that clade II initiated less host cell death during the acute stage of the infection. Viruses exploit essential cellular functions such as endocytosis, division, intracellular transport and apoptosis to achieve efficient replication. They extend their survival by mimicking apoptosis [42] and evading the host immune response, primarily by vesicular sequestration and the active subversion of innate immune responses [43]. Apoptotic mimicry has been shown to facilitate host cell infection, replication and immune evasion of flaviviruses, including DENV [42]. Therefore, it is plausible to assume that clade II viruses may possess a better ability to replicate in mammalian cells than clade I through a mechanism similar to apoptotic mimicry. The better replication ability of clade II causes potentially higher virus titers in human blood meals, which is likely to have facilitated the infection of *Ae. aegypti* more efficiently than clade I. In Singapore, the vector control program conducts fogging and/or misting during outbreaks to destroy the infectious pools of adult mosquitoes in disease clusters [44]. This approach reduces the probability of a vector’s survival. Assuming that there was no difference in the vector’s life expectancy during the periods of clades I and II presence, relatively short EIP and high virus titers in vector and mammalian cells could have a collective effect in boosting the transmission potential of clade II, contributing to the local outbreak in 2007.

DENV replication involves successful virus entry into host cells and the subsequent multiplication of genetic material and packaging. Virus genetics plays a critical role in these processes, together with the dissemination in *Ae. Aegypti* [45,46,47]. While structural proteins are primarily involved in DENV entry and packaging, nonstructural (NS) proteins and noncoding regions play a crucial role in the replication of genetic material [48,49,50,51,52,53] and immune evasion [54,55,56,57,58,59]. Therefore, the genetic differences between clades I and II could have contributed to the phenotype differences observed in vector and mammalian hosts. Of the nine amino acid substitutions that distinguish clades I and II, four are in the structural genes: one mutation in the envelope gene (E-I61V) and three in the membrane gene (M-V36I, A71T and T75A). The remaining five substitutions are distributed across the nonstructural polyprotein: NS1 (S80T), NS2A (T119S), NS4A (R22K) and NS5 (T638K and A648I/M). Among them, NS4A-R22K, NS5-T638K and NS5-A648I/M substitutions are unique to clade II. Of these, only M-A71T, M-T75A (hydrophobic to hydrophilic and vice versa) and NS5-T638K (polar to positively charged) substitutions are nonconservative. Importantly, M-T75A and M-A71T substitutions reside within the prM-E protein signalase cleavage sequence [48]. M-T75 residue is highly conserved in all genotypes of DENV-2 and is the cleavage site between prM and E proteins. The potential effect of prM-E protein cleavage site substitutions on the posttranslational processing and packaging of clade II is noteworthy in light of clade II’s ability to achieve higher viral titers in mammalian cells and *Ae. aegypti* than clade I. Moreover, the role of mutations in nonstructural proteins in mediating the enhanced replication of clade II viruses cannot be underestimated.

Besides coding regions, UTRs also host functional elements required for flavivirus replication and vector adaptation [30,60]. In our analyses, 5′ UTR was identical between clades I and II, but the 3′ UTR included three substitutions (A10274G, C10387T and T10389C) in the variable region. The variable region of DENV 3′ UTR includes five stem loop structures (SL-I to SL-V) [30,31]. Empirical data suggest that changes in the variable region may influence the potential of viruses to infect mammalian cells but unlikely to have any effect on mosquito cells [30,61]. This was supported by the comparable peak midgut virus titers achieved by both clades in *Ae. aegypti*. However, substitutions that affect the stability of SL-II and SL-IV have been shown to induce the production of sub-genomic flavivirus RNA, resulting in an altered host immune response in mammalian cells [32,62] and high salivary gland infection rates and infectious virus titers in *Ae. aegypti* [63]. As illustrated in Figure S1, the SL-IV structure was different between the two clades. A clade I-like SL-IV structure is conserved in the cosmopolitan genotype (all sequences related to clade I and the Indian subcontinent lineage). On the other hand, a clade II-like structure is conserved in the Asian II, American/Asian and cosmopolitan (all sequences related to clade II) genotypes of DENV-2. While the analysis implied that 3′ UTR loop structures are important from an evolutionary point of view as well, it is worthwhile to further investigate in order to determine whether these 3′ UTR changes also modulate the transmission potential of clades I and II.

## 5. Conclusions

In conclusion, it is plausible to postulate that a DENV-2 clade replacement event contributed to the escalation of cases in Singapore during 2007–2008. The association between the newly emerged virus (clade II) and the outbreak is explained, at least partially, by the enhanced transmission potential of clade II viruses, which demonstrated short EIP and high dissemination rate in *Ae. aegypti* as well as high virus titers in both mammalian cells and *Ae. aegypti*. The probability of a virus being transmitted by a mosquito is positively correlated with the quantity of the disseminated virus [47]. Our findings, therefore, collectively explain the eruption of an outbreak in 2007 even when the *Aedes* premise index (0.68%) was significantly lower (1.7–2.2% from 2002 to 2004) than that recorded during the preceding, non-outbreak years. The findings of the present study also support the hypothesis proposed by Lee and colleagues [4] that the aggressive vector control measures in Singapore could select for virus lineages with better fitness in *Ae. aegypti*. As proposed previously, our study emphasizes the notion that mosquito-driven selection plays an integral part of arbovirus evolution.

## Figures and Tables

**Table 1:** Details of DENV-2 isolates used in the study.

Isolate	Genbank Accession Number	Year Isolated	Lineage ^¶^
SGEHI(D2)0232Y06	JN851128	2006	Clade I
SG(EHI)D2/0866Y07	KP685236	2007	Clade II

^¶^ SGEHI(D2)0232Y06 belonged to the lineage circulating in 2006, before the emergence of clade II in 2007 that caused an outbreak in 2007–2008. Clade II formed a lineage that was distinguishable from clade I by a genetic signature of nine fixed amino acid substitutions (described in the text). Virus strains were selected for in vitro and in vivo experiments to represent each clade based on this genetic signature.

## Data Availability

Genomic sequences used in the phylogenetic analysis are available in GenBank database under the accession numbers stated in the Methods section.

## Supplementary Materials

The following supporting information can be downloaded at: https://www.mdpi.com/article/10.3390/microorganisms10061100/s1, Figure S1: Comparison of RNA secondary structures of the variable region of 3′UTR.

## References

[1] Bhatt S., Gething P.W., Brady O.J., Messina J.P., Farlow A.W., Moyes C.L., Drake J.M., Brownstein J.S., Hoen A.G., Sankoh O. (2013). The global distribution and burden of dengue. Nature.

[2] Castro M.C., Wilson M.E., Bloom D.E. (2017). Disease and economic burdens of dengue. Lancet Infect. Dis..

[3] Wilder-Smith A., Ooi E.E., Horstick O., Wills B. (2019). Dengue. Lancet.

[4] Lee K.S., Lo S., Tan S.S., Chua R., Tan L.K., Xu H., Ng L.C. (2012). Dengue virus surveillance in Singapore reveals high viral diversity through multiple introductions and in situ evolution. Infect. Genet. Evol..

[5] Hapuarachchi H.C., Koo C., Rajarethinam J., Chong C.S., Lin C., Yap G., Liu L., Lai Y.L., Ooi P.L., Cutter J. (2016). Epidemic resurgence of dengue fever in Singapore in 2013–2014: A virological and entomological perspective. BMC Infect. Dis..

[6] Rajarethinam J., Ang L.W., Ong J., Ycasas J., Hapuarachchi H.C., Yap G., Chong C.S., Lai Y.L., Cutter J., Ho D. (2018). Dengue in Singapore from 2004 to 2016: Cyclical Epidemic Patterns Dominated by Serotypes 1 and 2. Am. J. Trop. Med. Hyg..

[7] Koh B.K., Ng L.C., Kita Y., Tang C.S., Ang L.W., Wong K.Y., James L., Goh K.T. (2008). The 2005 dengue epidemic in Singapore: Epidemiology, prevention and control. Ann. Acad. Med. Singap..

[8] Ler T.S., Ang L.W., Yap G.S., Ng L.C., Tai J.C., James L., Goh K.T. (2011). Epidemiological characteristics of the 2005 and 2007 dengue epidemics in Singapore-similarities and distinctions. West. Pac. Surveill. Response J..

[9] Holmes E.C., Twiddy S.S. (2003). The origin, emergence and evolutionary genetics of dengue virus. Infect. Genet. Evol..

[10] Suzuki K., Phadungsombat J., Nakayama E.E., Saito A., Egawa A., Sato T., Rahim R., Hasan A., Lin M.Y., Takasaki T. (2019). Genotype replacement of dengue virus type 3 and clade replacement of dengue virus type 2 genotype Cosmopolitan in Dhaka, Bangladesh in 2017. Infect. Genet. Evol..

[11] Lambrechts L., Fansiri T., Pongsiri A., Thaisomboonsuk B., Klungthong C., Richardson J.H., Ponlawat A., Jarman R.G., Scott T.W. (2012). Dengue-1 virus clade replacement in Thailand associated with enhanced mosquito transmission. J. Virol..

[12] Quiner C.A., Parameswaran P., Ciota A.T., Ehrbar D.J., Dodson B.L., Schlesinger S., Kramer L.D., Harris E. (2014). Increased replicative fitness of a dengue virus 2 clade in native mosquitoes: Potential contribution to a clade replacement event in Nicaragua. J. Virol..

[13] Teoh B.T., Sam S.S., Tan K.K., Johari J., Shu M.H., Danlami M.B., Abd-Jamil J., MatRahim N., Mahadi N.M., AbuBakar S. (2013). Dengue virus type 1 clade replacement in recurring homotypic outbreaks. BMC Evol. Biol..

[14] Kotaki T., Yamanaka A., Mulyatno K.C., Churrotin S., Labiqah A., Sucipto T.H., Soegijanto S., Kameoka M., Konishi E. (2014). Continuous dengue type 1 virus genotype shifts followed by co-circulation, clade shifts and subsequent disappearance in Surabaya, Indonesia, 2008–2013. Infect. Genet. Evol..

[15] Lee K.S., Lai Y.L., Lo S., Barkham T., Aw P., Ooi P.L., Tai J.C., Hibberd M., Johansson P., Khoo S.P. (2010). Dengue virus surveillance for early warning, Singapore. Emerg. Infect. Dis..

[16] Weaver S.C., Vasilakis N. (2009). Molecular evolution of dengue viruses: Contributions of phylogenetics to understanding the history and epidemiology of the preeminent arboviral disease. Infect. Genet. Evol..

[17] Hapuarachchi H.C., Koo C., Kek R., Xu H., Lai Y.L., Liu L., Kok S.Y., Shi Y., Chuen R.L., Lee K.S. (2016). Intra-epidemic evolutionary dynamics of a Dengue virus type 1 population reveal mutant spectra that correlate with disease transmission. Sci. Rep..

[18] Koo C., Tien W.P., Xu H., Ong J., Rajarethinam J., Lai Y.L., Ng L.C., Hapuarachchi H.C. (2018). Highly Selective Transmission Success of Dengue Virus Type 1 Lineages in a Dynamic Virus Population: An Evolutionary and Fitness Perspective. iScience.

[19] Lambrechts L., Chevillon C., Albright R.G., Thaisomboonsuk B., Richardson J.H., Jarman R.G., Scott T.W. (2009). Genetic specificity and potential for local adaptation between dengue viruses and mosquito vectors. BMC Evol. Biol..

[20] Tan L.K., Low S.L., Lam S., Teo D., Ng L.C. (2012). Evaluation of pathogen reduction systems to inactivate dengue and chikungunya viruses in apheresis platelets suspended in plasma. Int. J. Infect. Dis..

[21] Koo C., Nasir A., Hapuarachchi H.C., Lee K.S., Hasan Z., Ng L.C., Khan E. (2013). Evolution and heterogeneity of multiple serotypes of Dengue virus in Pakistan, 2006–2011. Virol. J..

[22] Hall T.A. (1999). BioEdit: A user-friendly biological sequence alignment editor and analysis program for Windows 95/98/NT. Nucleic Acids Symp. Ser..

[23] Kumar S., Stecher G., Tamura K. (2016). MEGA7: Molecular Evolutionary Genetics Analysis Version 7.0 for Bigger Datasets. Mol. Biol. Evol..

[24] Zuker M. (2003). Mfold web server for nucleic acid folding and hybridization prediction. Nucleic Acids Res..

[25] Li M.I., Wong P.S., Ng L.C., Tan C.H. (2012). Oral susceptibility of Singapore Aedes (Stegomyia) aegypti (Linnaeus) to Zika virus. PLoS Negl. Trop. Dis..

[26] Wong P.S., Li M.Z., Chong C.S., Ng L.C., Tan C.H. (2013). Aedes (Stegomyia) albopictus (Skuse): A potential vector of Zika virus in Singapore. PLoS Negl. Trop. Dis..

[27] Higgs S., Olson K.E., Kamrud K.I., Powers A.M., Beaty B., Beard C.B., Louis C. (1997). Viral Expression Systems and Viral Infections in Insects.

[28] Hierholzer J.C., Killington R.A. (1996). Virus Isolation and Quantitation.

[29] R Core Team (2014). R: A language and environment for statistical computing. R Foundation for Statistical Computing.

[30] Gebhard L.G., Filomatori C.V., Gamarnik A.V. (2011). Functional RNA elements in the dengue virus genome. Viruses.

[31] Ward A.M., Bidet K., Yinglin A., Ler S.G., Hogue K., Blackstock W., Gunaratne J., Garcia-Blanco M.A. (2011). Quantitative mass spectrometry of DENV-2 RNA-interacting proteins reveals that the DEAD-box RNA helicase DDX6 binds the DB1 and DB2 3′ UTR structures. RNA Biol..

[32] Chapman E.G., Moon S.L., Wilusz J., Kieft J.S. (2014). RNA structures that resist degradation by Xrn1 produce a pathogenic Dengue virus RNA. Elife.

[33] Klein E., Ben-Bassat H., Neumann H., Ralph P., Zeuthen J., Polliack A., Vanky F. (1976). Properties of the K562 cell line, derived from a patient with chronic myeloid leukemia. Int. J. Cancer.

[34] Diamond M.S., Roberts T.G., Edgil D., Lu B., Ernst J., Harris E. (2000). Modulation of Dengue virus infection in human cells by alpha, beta, and gamma interferons. J. Virol..

[35] Yu X., Cheng G. (2022). Adaptive Evolution as a Driving Force of the Emergence and Re-Emergence of Mosquito-Borne Viral Diseases. Viruses.

[36] Fontaine A., Lequime S., Moltini-Conclois I., Jiolle D., Leparc-Goffart I., Reiner R.C., Lambrechts L. (2018). Epidemiological significance of dengue virus genetic variation in mosquito infection dynamics. PLoS Pathog..

[37] Ko H.Y., Salem G.M., Chang G.J., Chao D.Y. (2020). Application of Next-Generation Sequencing to Reveal How Evolutionary Dynamics of Viral Population Shape Dengue Epidemiology. Front. Microbiol..

[38] Lequime S., Fontaine A., Ar Gouilh M., Moltini-Conclois I., Lambrechts L. (2016). Genetic Drift, Purifying Selection and Vector Genotype Shape Dengue Virus Intra-host Genetic Diversity in Mosquitoes. PLoS Genet..

[39] Lambrechts L., Lequime S. (2016). Evolutionary dynamics of dengue virus populations within the mosquito vector. Curr. Opin. Virol..

[40] Stica C.J., Barrero R.A., Murray R.Z., Devine G.J., Phillips M.J., Frentiu F.D. (2022). Global Evolutionary History and Dynamics of Dengue Viruses Inferred from Whole Genome Sequences. Viruses.

[41] Zhang C., Mammen M.P., Chinnawirotpisan P., Klungthong C., Rodpradit P., Monkongdee P., Nimmannitya S., Kalayanarooj S., Holmes E.C. (2005). Clade replacements in dengue virus serotypes 1 and 3 are associated with changing serotype prevalence. J. Virol..

[42] Amara A., Mercer J. (2015). Viral apoptotic mimicry. Nat. Rev. Microbiol..

[43] Green A.M., Beatty P.R., Hadjilaou A., Harris E. (2014). Innate immunity to dengue virus infection and subversion of antiviral responses. J. Mol. Biol..

[44] Sim S., Ng L.C., Lindsay S.W., Wilson A.L. (2020). A greener vision for vector control: The example of the Singapore dengue control programme. PLoS Negl. Trop. Dis..

[45] Fansiri T., Fontaine A., Diancourt L., Caro V., Thaisomboonsuk B., Richardson J.H., Jarman R.G., Ponlawat A., Lambrechts L. (2013). Genetic mapping of specific interactions between Aedes aegypti mosquitoes and dengue viruses. PLoS Genet..

[46] Filomatori C.V., Carballeda J.M., Villordo S.M., Aguirre S., Pallares H.M., Maestre A.M., Sanchez-Vargas I., Blair C.D., Fabri C., Morales M.A. (2017). Dengue virus genomic variation associated with mosquito adaptation defines the pattern of viral non-coding RNAs and fitness in human cells. PLoS Pathog..

[47] Lambrechts L. (2011). Quantitative genetics of Aedes aegypti vector competence for dengue viruses: Towards a new paradigm?. Trends Parasitol..

[48] Chambers T.J., Hahn C.S., Galler R., Rice C.M. (1990). Flavivirus genome organization, expression, and replication. Annu. Rev. Microbiol..

[49] Pena J., Harris E. (2011). Dengue virus modulates the unfolded protein response in a time-dependent manner. J. Biol. Chem..

[50] Yu C.Y., Hsu Y.W., Liao C.L., Lin Y.L. (2006). Flavivirus infection activates the XBP1 pathway of the unfolded protein response to cope with endoplasmic reticulum stress. J. Virol..

[51] Heaton N.S., Perera R., Berger K.L., Khadka S., Lacount D.J., Kuhn R.J., Randall G. (2010). Dengue virus nonstructural protein 3 redistributes fatty acid synthase to sites of viral replication and increases cellular fatty acid synthesis. Proc. Natl. Acad. Sci. USA.

[52] Panyasrivanit M., Khakpoor A., Wikan N., Smith D.R. (2009). Co-localization of constituents of the dengue virus translation and replication machinery with amphisomes. J. Gen. Virol..

[53] Mateo R., Nagamine C.M., Spagnolo J., Mendez E., Rahe M., Gale M., Yuan J., Kirkegaard K. (2013). Inhibition of cellular autophagy deranges dengue virion maturation. J. Virol..

[54] Kakumani P.K., Ponia S.S., S R.K., Sood V., Chinnappan M., Banerjea A.C., Medigeshi G.R., Malhotra P., Mukherjee S.K., Bhatnagar R.K. (2013). Role of RNA interference (RNAi) in dengue virus replication and identification of NS4B as an RNAi suppressor. J. Virol..

[55] Yu C.Y., Chang T.H., Liang J.J., Chiang R.L., Lee Y.L., Liao C.L., Lin Y.L. (2012). Dengue virus targets the adaptor protein MITA to subvert host innate immunity. PLoS Pathog..

[56] Aguirre S., Maestre A.M., Pagni S., Patel J.R., Savage T., Gutman D., Maringer K., Bernal-Rubio D., Shabman R.S., Simon V. (2012). DENV inhibits type I IFN production in infected cells by cleaving human STING. PLoS Pathog..

[57] Rodriguez-Madoz J.R., Belicha-Villanueva A., Bernal-Rubio D., Ashour J., Ayllon J., Fernandez-Sesma A. (2010). Inhibition of the type I interferon response in human dendritic cells by dengue virus infection requires a catalytically active NS2B3 complex. J. Virol..

[58] Munoz-Jordan J.L., Laurent-Rolle M., Ashour J., Martinez-Sobrido L., Ashok M., Lipkin W.I., Garcia-Sastre A. (2005). Inhibition of alpha/beta interferon signaling by the NS4B protein of flaviviruses. J. Virol..

[59] Ashour J., Laurent-Rolle M., Shi P.Y., Garcia-Sastre A. (2009). NS5 of dengue virus mediates STAT2 binding and degradation. J. Virol..

[60] Gritsun T.S., Gould E.A. (2007). Origin and evolution of flavivirus 5′UTRs and panhandles: Trans-terminal duplications?. Virology.

[61] Alvarez D.E., De Lella Ezcurra A.L., Fucito S., Gamarnik A.V. (2005). Role of RNA structures present at the 3′UTR of dengue virus on translation, RNA synthesis, and viral replication. Virology.

[62] Manokaran G., Finol E., Wang C., Gunaratne J., Bahl J., Ong E.Z., Tan H.C., Sessions O.M., Ward A.M., Gubler D.J. (2015). Dengue subgenomic RNA binds TRIM25 to inhibit interferon expression for epidemiological fitness. Science.

[63] Pompon J., Manuel M., Ng G.K., Wong B., Shan C., Manokaran G., Soto-Acosta R., Bradrick S.S., Ooi E.E., Misse D. (2017). Dengue subgenomic flaviviral RNA disrupts immunity in mosquito salivary glands to increase virus transmission. PLoS Pathog..

