# Supplementary material for: Lineage Replacement Associated with Fitness Gain in Mammalian Cells and Aedes aegypti: A Catalyst for Dengue Virus Type 2 Transmission
Source: Microorganisms. 2022 May 26;10(6):1100. doi: 10.3390/microorganisms10061100 (PMC9231088; doi:10.3390/microorganisms10061100)
Supplement: Supplementary file 1 [file microorganisms-10-01100-s001.zip › microorganisms-1736486 - supplementary.pdf]

Lineage replacement associated with fitness gain in mammalian cells and Aedes aegypti: A catalyst for dengue virus type 2 transmission

Cheong Huat Tan[1£], Li Kiang Tan[1£], Hapuarachchige Chanditha Hapuarachchi[1£*], Pei Sze Jeslyn Wong[1], Mei Zhi Irene Li[1], Wing Yan Wong[1], Lee Ching Ng[1,2*]

[*]Corresponding authors

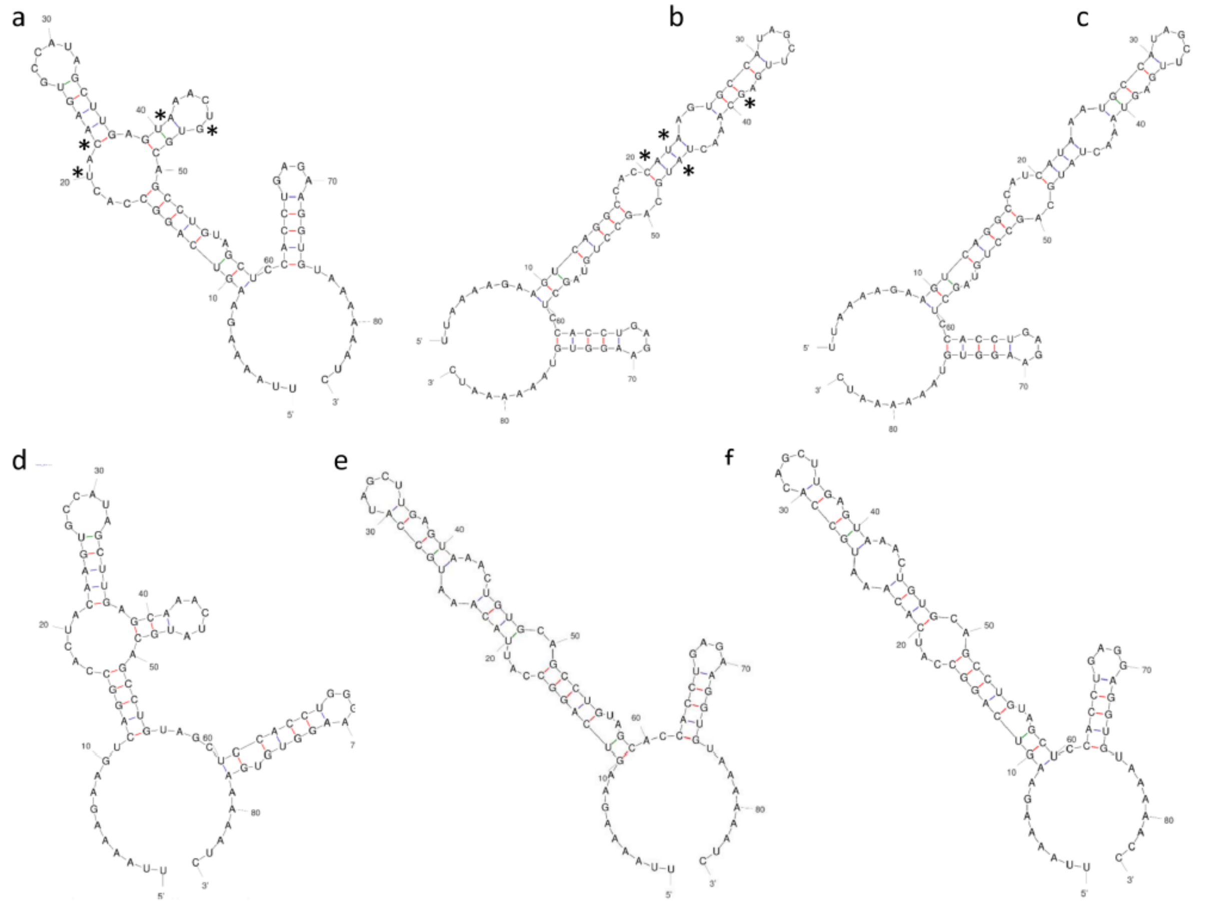

Figure S1. Comparison of RNA secondary structures of the variable region of 3′UTR.

The complete sequence of 3′UTR was used to draw the secondary structure as predicted

by the MFOLD web server [21]. Only the region predicted to be structurally different between clades I and II viruses is shown. Positions shown correspond to respective nucleotides from the beginning of 3′UTR. (a). Clade I (SGEHI(D2)0232Y06) (b). Clade II (SG(EHI)D2/0866Y07) (c). DENV-2 Asian I genotype (GenBank accession no. NC001474) (d). DENV-2 cosmopolitan genotype – Indian sub-continent lineage (e). DENV-2 Asian II genotype (GenBank accession no. AF204178) and (f). DENV-2 American/Asian genotype (GenBank accession no. AF208496). Asian I genotype was not included as the structure was vastly different. The 3′ UTR sequences of American genotype were not available in the GenBank database. Substitutions in clade I and II viruses are indicated with asterisks. Stem loop structures were named as illustrated elsewhere [26,27].
